# Changes in emergency department visits after introducing a triage and primary care consultation telephone service

**DOI:** 10.1186/1757-7241-23-S1-A19

**Published:** 2015-07-16

**Authors:** Jakob L Forberg, Torben B Højmark, Leif P Jensen

**Affiliations:** 1 Department of Emergency Medicine, Hospital of Northern Zealand, Hilleroed, Denmark; 2 Department of Health Data and Analysis, Hospital of Northern Zealand, Hilleroed, Denmark; 3 Board of Directors, Hospital of Northern Zealand, Hilleroed, Denmark

## Background

In January 2014 the Capital Region introduced a single point of contact telephone service (1813) for all acute and non-emergent injuries and illnesses. In case of an acute injury, citizens are instructed to call 1813 (24/7) before attending the Emergency Department (ED). For telephone consultation or ED referral due to acute illness, citizens are instructed to call 1813 after general practice (GP) opening hours. Changes of ED visits in the early implementation phase were studied.

## Method

ED visits at Hospital of Northern Zealand from the period February 1 to June 30 in 2013 and 2014 were compared. Data were retrospectively extracted from the patient administrative system. For Hillerød ED data was in addition extracted from the ED information system Cetrea. Out of hours primary care consultations at the hospital were not included. All patients with injuries were seen in the ED. Children less than 17 years of age with illness are treated in the paediatric ED and, thus, not included in the study.

## Results

There was an overall increase in ED admissions from 16,277 to 17,456. Admissions during GP opening hours and out of hours, increased with 4.8% and 9.0%, respectively. Patient transfers between specialties after admissions decreased significantly from 8.9% to 7.7%. Ambulatory ED visits increased from 26,908 to 27,819. Visits due to orthopaedic injuries in Hillerød ED increased by 2%. The distribution of arrival time for acute ambulatory visits was significantly changed. The amount of patients arriving at 10-13 o'clock decreased and the amount at 21-23 o'clock increased.

## Conclusions

In the early implementation phase of the 1813 service ambulatory and orthopaedic ED visits had not yet decreased. The amount of admissions increased. The rise in admissions was higher during the hours when 1813 mainly refers to the ED. A rise in admission during GP opening hours contributed to the overall increase. The arrival rate during peak arrival hours was slightly reduced. In order to reduce ED crowding further effort is needed to reduce low risk ED visits and to match the arrival rate of low risk patients with ED capacity and available competencies.

